# Origins of ETP leukemia

**DOI:** 10.18632/oncoscience.456

**Published:** 2018-08-22

**Authors:** Christopher A. G. Booth, Sten Eirik W. Jacobsen, Adam J. Mead

**Affiliations:** MRC Molecular Haematology Unit, Weatherall Institute of Molecular Medicine, University of Oxford, John Radcliffe Hospital, Headington, Oxford, OX3 9DS UK

**Keywords:** leukemia, early thymic progenitor, BET Inhibition, ETP-ALL, leukemic stem cells

The relationship between the identity of the normal cells targeted by oncogenic mutations and the phenotype of the resulting cancer is not well understood. In a recent study [1], we sought to address this question by modelling a distinct and chemotherapy-resistant subtype of T cell acute lymphoblastic leukemia (T-ALL), known as early thymic progenitor (ETP) leukemia.

ETPs are the most primitive T cell progenitor population found in the thymus, and unlike down-stream thymocyte progenitors, they retain myeloid as well as B cell potential [2]. Moreover, unlike hematopoietic stem cells (HSCs), ETPs do not sustain extensive self-renewal potential, and must therefore be continually replenished by thymus-seeding progenitors from the bone marrow.

The existence of ETP leukemia was proposed in 2009 based on distinct clinical features combined with gene expression profiling [3], demonstrating an expression signature distinct from other T-ALLs and reminiscent of mouse ETPs, including increased HSC and myeloid lineage-associated gene expression. This raised the interesting possibility that these leukemias could have originated from oncogenic transformation of ETPs.

A landmark study published in 2012 profiled the mutational landscape and gene expression of 64 ETP leukemias [4]. EZH2 and RUNX1 inactivating mutations were both common and significantly associated in these patients, similar to what is observed in myeloid malignancies [5]. We decided to explore the possible functional relevance of this association in ETP leukemias by specifically targeting inactivation of these two transcriptional regulators to early lymphoid progenitors in mice.

While mice with combined Ezh2 and Runx1 loss of function mutations targeted to early lymphoid progenitors (DKO mice) failed to develop leukemia and showed dramatically reduced thymus cellularity, the numbers of ETPs in the thymus were selectively and markedly expanded. Gene expression profiling of these cells using global RNA-sequencing revealed upregulation of HSC, myeloid and signaling-associated transcription, all characteristic features of ETP leukemia. Moreover, single-cell gene expression analysis of mutated ETPs showed that the upregulated HSC and myeloid genes were co-expressed with lymphoid genes within individual ETPs.

Another intriguing finding in ETP leukemias is the high prevalence of activating mutations in RAS signalling pathway components [4], mutations more commonly associated with acute myeloid leukemias (AMLs) than T-ALLs. In fact, internal tandem duplication (ITD) activating mutations of the cytokine receptor FLT3, common in AMLs, were shown among T-ALLs to be exclusively present in ETP leukemia patients. Upon introduction of a constitutively activating Flt3-ITD mutation together with the Ezh2 and Runx1 loss of function mutations in early lymphoid progenitors (DKOITD mice), mice rapidly succumbed to an aggressive acute leukemia, which showed gene expression of both the myeloid and T cell lineages, and an overall expression profile closely mirroring that of human ETP leukemia.

The expansion of the ETP population observed in both DKO and DKOITD mice, combined with the ETP-like gene expression of DKOITD leukemias, prompted us to test whether DKOITD ETPs might have acquired self-renewal potential, and thus act as disease-propagating leukemia stem cells (LSCs). To do this we FACS-sorted ETPs as well as other T cell progenitor populations from thymuses of DKOITD mice and transplanted them into wild-type recipient mice. Notably, unlike wild type ETPs or mutated down-stream thymic progenitors, ETPs were able to engraft and generate the same leukemia phenotype in recipient mice, providing experimental evidence that ETPs can indeed undergo oncogenic transformation to become LSCs.

Using this novel ETP leukemia mouse model to explore potential therapeutic strategies for this chemotherapy-resistant cancer, we found that inactivation of Ezh2 in DKOITD bone marrow drives upregulation of gene expression via loss of H3K27me3 and gain of H3K27ac, as expected given the role of EZH2 as part of the polycomb repressive complex 2 (PRC2) [6]. In agreement with a previous study using malignant peripheral nerve sheath tumors [7], this conferred sensitivity to the BET inhibitors JQ1 and I-BET151 both *in vitro* and *in vivo*. Strikingly, human PRC2-mutant ETP leukemia xenograft samples also showed very high sensitivity to JQ1 treatment *in vivo*, completely halting the growth of one patient leukemia in NSG recipient mice even as a single agent. These findings implicate BET inhibition as a potential therapeutic strategy for PRC2-inactivated ETP leukemias, and justify further evaluation of these compounds in other PRC2-deficient malignancies.

Our study [1] provides experimental evidence that the phenotypic and gene expression profiles of a cancer can be traced back to those of the cell of origin. By targeting relevant mutations specifically to early lymphoid progenitors retaining myeloid differentiation potential, we generated leukemias displaying a biphenotypic myeloid/T lymphoid phenotype. Importantly, for the first time since the identification of human ETP leukemia [3], we confirmed that ETPs do indeed possess the potential to become LSCs upon introduction of recurrent ETP leukemia associated mutations, and demonstrated how this insight can be exploited in the development of novel cancer therapies.

## References

[1] Booth CAG (2018). Cancer Cell.

[2] Luc S (2012). Nat Immunol.

[3] Coustan-Smith E (2009). Lancet Oncol.

[4] Zhang J (2012). Nature.

[5] Papaemmanuil E (2013). Blood.

[6] Margueron R (2011). Nature.

[7] De Raedt T (2014). Nature.

